# Prokinetics for the treatment of functional dyspepsia: Bayesian network meta-analysis

**DOI:** 10.1186/s12876-017-0639-0

**Published:** 2017-06-26

**Authors:** Young Joo Yang, Chang Seok Bang, Gwang Ho Baik, Tae Young Park, Suk Pyo Shin, Ki Tae Suk, Dong Joon Kim

**Affiliations:** Department of Internal Medicine, Hallym University College of Medicine, Chuncheon Sacred Heart Hospital, Sakju-ro 77, Chuncheon, Gangwon-do 24253 Republic of Korea

**Keywords:** Comparative effectiveness research, Functional dyspepsia, Network meta-analysis, Systematic review, Prokinetics

## Abstract

**Background:**

Controversies persist regarding the effect of prokinetics for the treatment of functional dyspepsia (FD). This study aimed to assess the comparative efficacy of prokinetic agents for the treatment of FD.

**Methods:**

Randomized controlled trials (RCTs) of prokinetics for the treatment of FD were identified from core databases. Symptom response rates were extracted and analyzed using odds ratios (ORs). A Bayesian network meta-analysis was performed using the Markov chain Monte Carlo method in WinBUGS and NetMetaXL.

**Results:**

In total, 25 RCTs, which included 4473 patients with FD who were treated with 6 different prokinetics or placebo, were identified and analyzed. Metoclopramide showed the best surface under the cumulative ranking curve (SUCRA) probability (92.5%), followed by trimebutine (74.5%) and mosapride (63.3%). However, the therapeutic efficacy of metoclopramide was not significantly different from that of trimebutine (OR:1.32, 95% credible interval: 0.27–6.06), mosapride (OR: 1.99, 95% credible interval: 0.87–4.72), or domperidone (OR: 2.04, 95% credible interval: 0.92–4.60). Metoclopramide showed better efficacy than itopride (OR: 2.79, 95% credible interval: 1.29–6.21) and acotiamide (OR: 3.07, 95% credible interval: 1.43–6.75). Domperidone (SUCRA probability 62.9%) showed better efficacy than itopride (OR: 1.37, 95% credible interval: 1.07–1.77) and acotiamide (OR: 1.51, 95% credible interval: 1.04–2.18).

**Conclusions:**

Metoclopramide, trimebutine, mosapride, and domperidone showed better efficacy for the treatment of FD than itopride or acotiamide. Considering the adverse events related to metoclopramide or domperidone, the short-term use of these agents or the alternative use of trimebutine or mosapride could be recommended for the symptomatic relief of FD.

**Electronic supplementary material:**

The online version of this article (doi:10.1186/s12876-017-0639-0) contains supplementary material, which is available to authorized users.

## Background

Functional dyspepsia (FD) is a common condition in clinical practice [1]. According to the Rome III and IV criteria, FD is defined as the presence of at least one of the following symptoms; postprandial fullness, early satiation, epigastric pain or burning, without evidence of structural disease to explain the symptoms fulfilling time criteria for the last three months with symptom onset at least six months before diagnosis and a frequency of at least three days per week [2, 3]. FD is subcategorized into two distinct conditions, which are postprandial distress syndrome, associated with meal-induced bothersome fullness or early satiation, and epigastric pain syndrome, showing bothersome epigastric pain or burning [2, 3].

This condition involves complex pathophysiologic mechanisms and shares overlapping symptoms with gastroesophageal reflux disease or other functional gastrointestinal disorders. The mainstay of the treatment of FD has been to target gastric acid secretion and impaired gut motility. The role of acid inhibitory drugs in the treatment of FD is well established [4]. In a subtype of FD, the response rate of epigastric pain syndrome to acid inhibitory therapy is known to be better than that of post-prandial distress syndrome [5].

Excluding acid inhibitory therapy, prokinetics are the mainstay of the treatment of FD. However, the role of prokinetics has not been well established, and inconsistent results have been reported regarding the therapeutic efficacy of each drug [6–10]. Moreover, previously published pairwise meta-analyses were conducted by examining several drugs with different mechanisms of action as a single group, which cannot present the efficacy of each prokinetic agent [8, 9]. However, head-to-head efficacy comparison of prokinetic agents in the treatment of FD is not easy and pooled comparative efficacy has not been established.

Unlike traditional pair-wise meta-analysis, network meta-analysis enables comparing more than 2 treatments strengthening the precision in the estimate and presenting relative effect sizes in a rank order [11]. Therefore, this study aimed to evaluate the comparative effectiveness of each prokinetic in the treatment of FD using an indirect comparison method.

## Methods

### Literature search

A systematic review was conducted using electronic databases. MEDLINE (PubMed), EMBASE, and the Cochrane Central Register of Controlled Trials (CENTRAL) in the Cochrane Library were searched using common keywords related to FD and prokinetics (inception to July 2015). The keywords were as follows: ‘functional dyspepsia’, ‘prokinetics’, ‘mosapride’, ‘itopride’, ‘trimebutine’, ‘metoclopramide’, ‘domperidone’, and ‘acotiamide’, drawn from MeSH or Emtree terminology and using Boolean operators. Only publications involving human subjects were searched. The bibliographies of relevant articles were also reviewed to identify additional studies. The language of publication was not restricted and all publications except Korean and English were translated using commercial translation service.

### Selection criteria

We included only randomized controlled trials (RCTs) meeting all of the following criteria: 1) designed to evaluate FD in the target or control group; 2) included a group that was given prokinetics and a comparison group that was given placebo or other prokinetics; and 3) presented comparative outcomes about symptomatic relief rates of FD after treatment. Exclusion criteria were as follows: 1) incomplete data or 2) review article.

### Selection of relevant studies

Two of the authors (C.S.B. and G.H.B.) independently evaluated the eligibility of all studies retrieved from the databases based on the predetermined selection criteria. The abstracts of all identified studies were reviewed to exclude irrelevant articles. Full-text review was performed to determine whether the inclusion criteria were satisfied by the remaining studies. Disagreements between the two evaluators were resolved by discussion or by consultation with a third author (D.J.K.).

### Assessment of methodological quality

The methodological quality of the enrolled studies was assessed using the Risk of Bias table (RoB). The RoB was assessed as described in the Cochrane handbook by recording the method used to generate the randomization sequence, allocation concealment, the determination of whether blinding was implemented for participants or staff, and whether there was evidence of selective reporting of the outcomes [12]. Review Manager version 5.3.3 (Revman for Windows 7, the Nordic Cochrane Centre, Copenhagen, Denmark) was used to generate the RoB table. Two of the authors (C.S.B. and G.H.B.) independently evaluated the methodological quality of all studies, and any disagreements between the two evaluators were resolved by discussion or by consultation with a third author (D.J.K.).

### Statistical analysis

We investigated the efficacy of prokinetics for the treatment of FD using odds ratios (ORs). We calculated the ORs based on an intention-to-treat analysis, when possible, from the original articles to compare the efficacy of prokinetics for the treatment of FD. Network meta-analyses were conducted using the Bayesian Markov Chain Monte Carlo method in WinBUGS 1.4.3 (MRC Biostatistics Unit, Cambridge, and Imperial College School of Medicine, London, UK) and Microsoft-Excel-based network meta-analysis tool (NetMetaXL) [11]. Effect sizes for the Bayesian network meta-analysis were described with 95% credible interval. Statistical validity is guaranteed when the 95% credible interval does not include 1. The detailed data input form and initial data for the analysis of network meta-analysis can be found in the Additional file 1.

## Results

### Identification of relevant studies

**Fig. 1:**
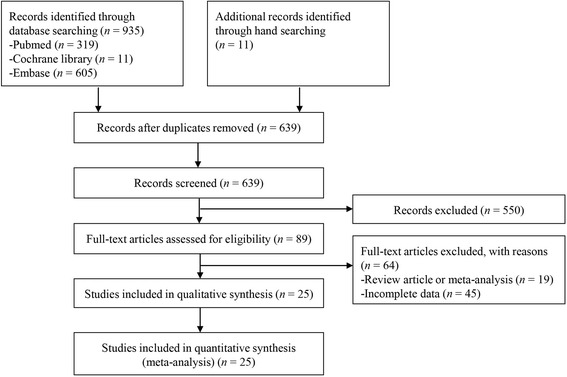
Flow diagram for identification of relevant studies

Figure 1 shows a flow diagram of how relevant studies were identified. A total of 946 articles were identified by searching 3 core databases and by hand searching the relevant bibliographies. In all, 307 duplicate articles and an additional 550 articles were excluded during the initial screening after reviewing the titles and abstracts. The full texts of the remaining 89 articles were thoroughly reviewed. Among these studies, 63 were excluded from the final analysis. The reasons for the exclusion of studies during the final review were as follows: review article (*n* = 19) or incomplete data (*n* = 45). The remaining 25 RCTs were included in the final analysis.

### Characteristics of studies included in the final analysis

In the 25 RCTs, we identified a total of 4473 participants (1602 placebo, 955 itopride, 773 domperidone, 713 acotiamide, 335 mosapride, 68 metoclopramide, and 27 trimebutine-treated participants). The enrolled studies were published between 1978 and 2012 [13–37]. All of the articles were full-text format except 2 studies [13, 36], which was in abstract format. More than half of the enrolled studies were conducted in Asia (*n* = 14) [13–26], followed by 9 studies in Europe [27–34], and the remaining 3 studies in the US [35–37]. Sixteen English-, 8 Chinese-, 1 Portuguese-, and 1 Korean-language studies were enrolled. The treatment duration ranged from 2 to 12 weeks. Eighteen studies used control medication as a placebo, whereas the remaining 8 studies performed direct head-to-head comparisons of prokinetic agents. All the prokinetic agents were orally administered and the detailed dosage, duration of prokinetics, and characteristics of the enrolled studies are shown in Table 1.

**Table 1:** Clinical data of included studies

Treatment & study	Location (language)	Age (years)	Sex	Placebo	Itopride	Mosapride	Domperidone	Acotiamide	Metoclopramide	Trimebutine	Dosage & treatment duration
				Effective/Total	Effective/Total	Effective/Total	Effective/Total	Effective/Total	Effective/Total	Effective/Total	
Van Ganse W (1978)	Belgium (English)	mean 50 (range 24–82)	M: 42 F: 31 (2 drop out)	9/36			31/35				10 mg × 4/day, 2 weeks
Bekhti A (1979)	Belgium (English)	domperidone: median 43.5 (range 19–67), placebo: 47 (50–73)		4/20			13/20				10 mg × 4/day, 4 weeks
De Loose F (1979)	Belgium (English)	median 40 (range 19–63)	M: 16, F: 27 (multiphase study)	22/70			62/68				10 mg × 4/day, 2 weeks
De Loose (1979)	Belgium (English)	median 40 (range 19–63)	M: 16, F: 27 (multiphase study)	22/70					50/68		10 mg × 4/day, 2 weeks
Van Outryve M (1979)	Belgium (English)	domperidone: median 63 (range 32–80), placebo: 52 (25–81)	M: 16, F: 22	11/22			13/16				20 mg, × 3/day, 2 weeks
Van de Mierop L (1979)	Belgium (English)	median 56 (range 32–76)	M: 9, F: 23	2/15			12/17				10 mg, × 3/day, 4 weeks
Davis RH (1988)	US (English)	mean 30 (range 18–48)	M: 1, F: 15	3/7			7/9				20 mg, × 2/day, 6 weeks
Teixeira CR (2000)	Portugal (Portuguese)	39 ± 13, (mean ± SD), (range 18–74)	M: 19, F: 46 (22 cisapride allocated population)	5/16						18/27	200 mg, × 3/day, 15 days
Zhou LY (2000)	China (Chinese)	itopride: 42.6 ± 12.3, domperidone: 42.6 ± 12.8, (mean ± SD)	Itopride; M: 34. F: 66, Domperidone; M: 38, F: 63		79/100		74/101				itopride 50 mg, domperidone 10 mg × 3/day, 2 weeks
Hallerbäck BI (2002)	Europe (multicenter) (English)	range 18–75	Mosapride; M: 49, F: 94, Placebo; M: 44, F: 97	84/141		84/143					10 mg × 2/day, 6 weeks
Sun Jing (2003)	China (Chinese)	range 18–70			58/115		62/117				itopride 50 mg, domperidone 10 mg × 3/day, 2 weeks
Mo Jian-zhong (2003)	China (Chinese)	mean 47.39 (range 21–70)	M: 29, F: 51		38/39		29/40				itopride 50 mg, domperidone 10 mg × 3/day, 2 weeks
Chen Xi (2004)	China (Chinese)	itopride: 34.7 ± 8.9, domperidone: 36.3 ± 11.1, (mean ± SD)			17/20		14/20				itopride 50 mg, domperidone 10 mg × 3/day, 4 weeks
Amaranpukar DN (2004)	India (English)	itopride: 45.23 ± 13.07, mosapride: 39.79 ± 10.82, (mean ± SD)	M: 30, F: 30		28/30	19/30					itopride 50 mg, mosapride 5 mg × 3/day, 2 weeks
Chen Shi-yao (2004)	China (Chinese)	mosapride: 44 ± 12, domperidone: 43 ± 13, (mean ± SD),	M: 108, F: 123			106/118	92/113				mosapride 5 mg, domperidone 10 mg × 3/day, 4 weeks
Zhu Chang-Qing (2005)	China (Chinese)				77/119		73/117				itopride 50 mg, domperidone 10 mg × 3/day, 4 weeks
Li Yan-Hong (2005)	China (Chinese)	itopride: 38 ± 12, domperidone: 38 ± 12, (mean ± SD)	M: 94, F: 106		89/100		89/100				itopride 50 mg, domperidone 10 mg × 3/day, 4 weeks
Matsueda K (2005)	Japan (English)	mean 39	60% female	21/32				28/33			100 mg × 3/day, 4 weeks
Holtmann G (2006)	Germany (English)	47.9 ± 15.8, (mean ± SD)	63.5% female	56/136	75/128						100 mg × 3/day, 8 weeks
Talley NJ (2008)	US (English)			35/104				58/103			200 mg × 3/day, 12 weeks
Talley NJ (2008)	US (English)	itopride: 42.6 ± 12.8, placebo: 43.0 ± 12.5, (mean ± SD)	Itopride: female 64.6%, placebo: female 69.8%	112/316	115/304						100 mg × 3/day, 8 weeks
Lin Jinkun (2009)	China (Chinese)	range 19–65	M: 20, F: 40	12/30		25/30					5 mg × 3/day, 2 weeks
Yeon-Mi Kim (2010)	Korea (Korean)	mosapride: 29.79 ± 7.56, placebo: 31.86 ± 11.53, (mean ± SD)	M: 4, F: 24	5/14		10/14					5 mg × 3/day, 2 weeks
Matsueda K (2010)	Japan (English)	acotiamide: 37.5 ± 11.5, placebo: 37.3 ± 10.2, (mean ± SD)	M: 100, F: 111	43/107				52/104			100 mg × 3/day, 4 weeks
Kusunoki H (2012)	Japan (English)	acotiamide: 40.3 ± 13.2, placebo: 40.6 ± 13.0, (mean ± SD)	M: 13, F: 24 (5 drop out)	3/21				6/21			100 mg × 3/day, 14–18 days
Matsueda K (2012)	Japan (English)	acotiamide: 37.6 ± 10.7, placebo: 37.1 ± 9.9, (mean ± SD)	M: 363, F: 529 (5 drop out)	154/445				235/452			100 mg × 3/day, 4 weeks
Total				603/1602	576/955	244/335	571/773	379/713	50/68	18/27	

‘Effective/Total’ means number of symptom improved patients/total number of patients for given each prokinetic agent

**Fig. 2:**
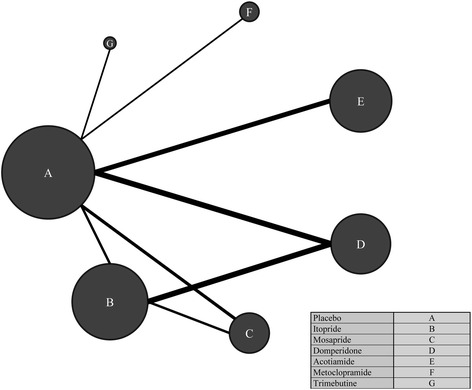
Network plot of relevant studies. Circles represent the each prokinetic drug as a node and lines represent the direct comparisons. The extent of circle indicates the number of included participants in each prokinetic drug and the line thickness indicates the number of studies included in each comparison

Figure 2 shows the network plot of relevant studies. Circles represent each prokinetic drug as a node and lines represent the direct comparisons. The extent of the circle indicates the number of included participants for each prokinetic drug, and the line thickness indicates the number of studies included in each comparison. Placebo was the biggest node, while the node size of metoclopramide and trimebutine was relatively smaller than the other remaining prokinetics. Direct comparisons were made between B (itopride) and D (domperidone) and between B (itopride) and C (mosapride). The remaining comparisons were performed in a pairwise manner.

### Comparative efficacy of prokinetics in FD

**Fig. 3:**
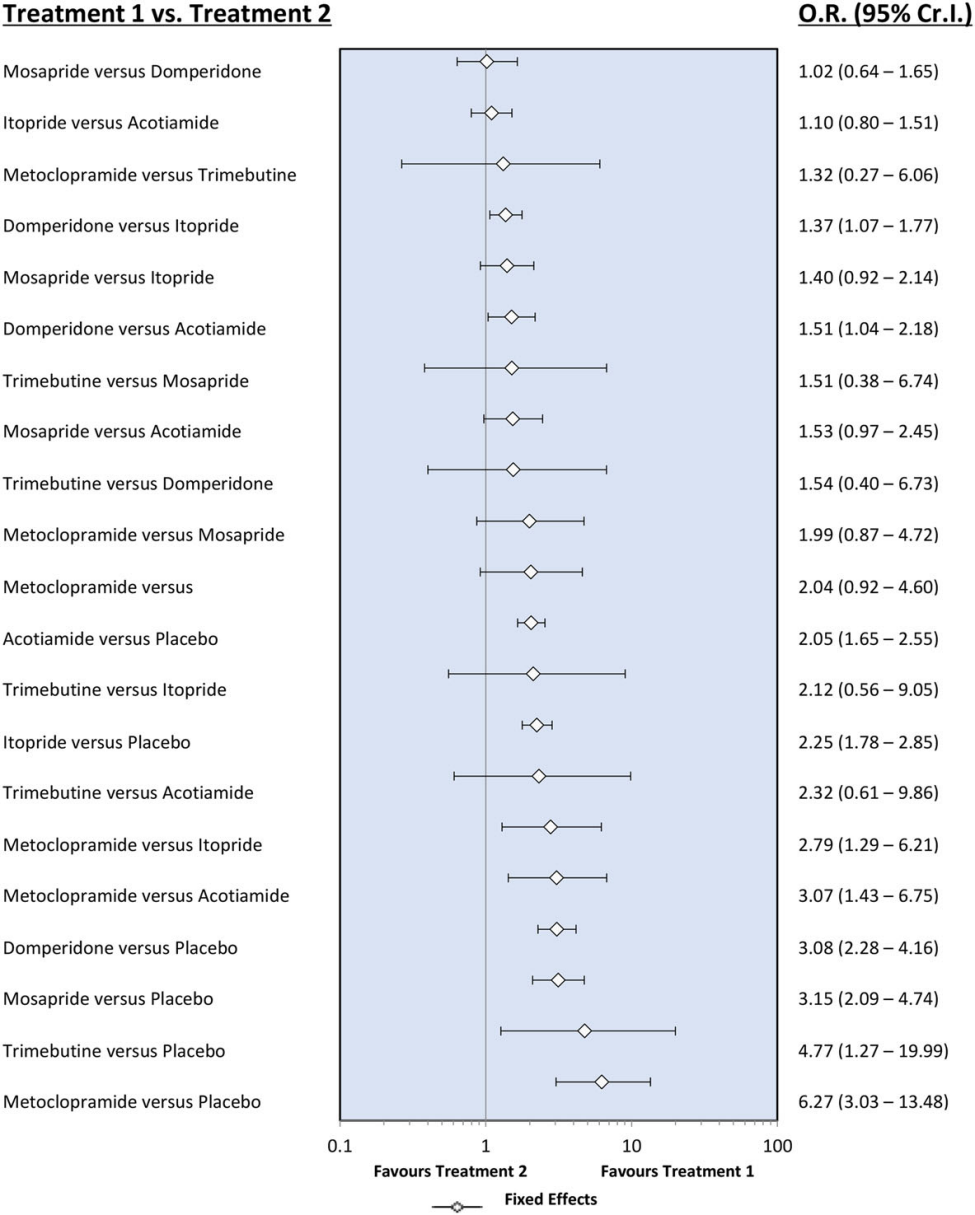
Forest plot of enrolled studies. Forest plot shows relative effect of each prokinetic agent. Diamond is the summary estimate from the pooled studies with 95% Cr. Cr: credible interval

Figure 3 shows the Forest plot of the results from a Bayesian network meta-analysis of the enrolled studies. The fixed-effect model was adopted based on the DIC statistics. The relative efficacy is plotted as OR with 95% credible interval. Based on these results, we calculated the surface under the cumulative ranking curve (SUCRA), which is the converted value reflecting the probability of a treatment being the best according to the ranking of each treatment [11]. Table 2 shows the SUCRA of each treatment regimen. A higher SUCRA value indicates better therapeutic results based on the indirect comparison method [38]. Metoclopramide showed the best SUCRA probability (92.5%), followed by trimebutine (74.5%), mosapride (63.3%), domperidone (62.9%), itopride (32.4%), acotiamide (24.3%), and placebo.

**Table 2:** SUCRA of each treatment regimen

Treatment	^a^SUCRA
Metoclopramide	0.925
Trimebutine	0.745
Mosapride	0.633
Domperidone	0.629
Itopride	0.324
Acotiamide	0.243
Placebo	0.002

^a^SUCRA, surface under the cumulative ranking curve

However, the therapeutic efficacy of metoclopramide was not significantly different from that of trimebutine (OR: 1.32, 95% credible interval: 0.27–6.06), mosapride (OR: 1.99, 95% credible interval: 0.87–4.72), and domeperidone (OR: 2.04, 95% credible interval: 0.92–4.60) in the league table, which shows the relative efficacy using OR and 95% credible interval (Table 3). Metoclopramide showed better efficacy than itopride (OR: 2.79, 95% credible interval: 1.29–6.21) and acotiamide (OR: 3.07, 95% credible interval: 1.43–6.75). Domperidone also showed better efficacy than itopride (OR: 1.37, 95% credible interval: 1.07–1.77) and acotiamide (OR: 1.51, 95% credible interval: 1.04–2.18).

**Table 3:** League table of each treatment regimen

Metoclopramide						
1.32 (0.27–6.06)	Trimebutine					
1.99 (0.87–4.72)	1.51 (0.38–6.74)	Mosapride				
2.04 (0.92–4.60)	1.54 (0.40–6.73)	1.02 (0.64–1.65)	Domperidone			
2.79 (1.29–6.21)	2.12 (0.56–9.05)	1.40 (0.92–2.14)	1.37 (1.07–1.77)	Itopride		
3.07 (1.43–6.75)	2.32 (0.61–9.86)	1.53 (0.97–2.45)	1.51 (1.04–2.18)	1.10 (0.80–1.51)	Itopride	
6.27 (3.03–13.48)	4.77 (1.27–19.99)	3.15 (2.09–4.74)	3.15 (2.09–4.74)	2.25 (1.78–2.85)	2.05 (1.65–2.55)	Placebo

Odds ratio with 95% credible interval is described in each column. Prokinetic agent in the top left means better efficacy and statistical validity is guaranteed when the 95% credible interval does not include 1

**Fig. 4:**
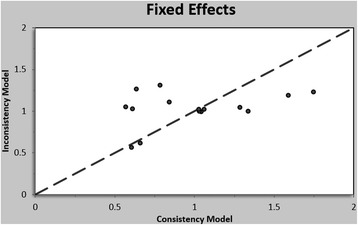
Inconsistency plot of enrolled studies. Plot of the posterior mean deviance of each study for the consistency model (horizontal axis), and the unrelated mean-effects model (vertical axis), along with the line of equality

Figure 4 shows the inconsistency plot of the enrolled studies. The plot demonstrates the posterior mean deviance of each study for the consistency model (horizontal axis), and the unrelated mean-effects model (vertical axis), along with the line of equality. There is some probability of inconsistency in the plot.

### Adverse events related to prokinetics

Adverse events related to prokinetics were as follows; domperidone induced diarrhea, constipation, intestinal colic, galactorrhea, bilateral breast tenderness, hyperprolactinemia, headache, dizziness, insomnia, and skin scare [19, 28, 35]; mosapride induced diarrhea, constipation, abdomen pain, dry mouth, fatigue, dizziness, headache, leg pain, and nausea [33]; itopride induced abdomen pain, diarrhea, constipation, nausea, and hyperprolactinemia [34, 38]; Acotiamide induced headache, diarrhea, increase in serum alanine aminotransferase, potassium, triglycerides, γ-glutamyltransferase, nasopharyngitis, and hyperprolactinemia [24–26]. Even control groups using placebo showed similar adverse events with treatment group taking prokinetics. Most adverse events were mild to moderate and have resolved after discontinuing prokinetics.

### Methodological quality

**Fig. 5:**
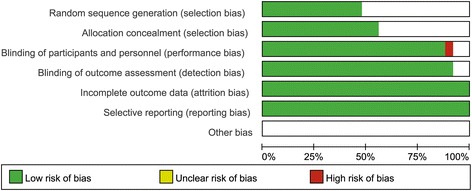
Summary of risk of bias. This figure summarizes the risk of bias for each study as a risk of bias summary of the overall meta-analysis. Green represents low risk of bias and red represents high risk of bias

**Fig. 6:**
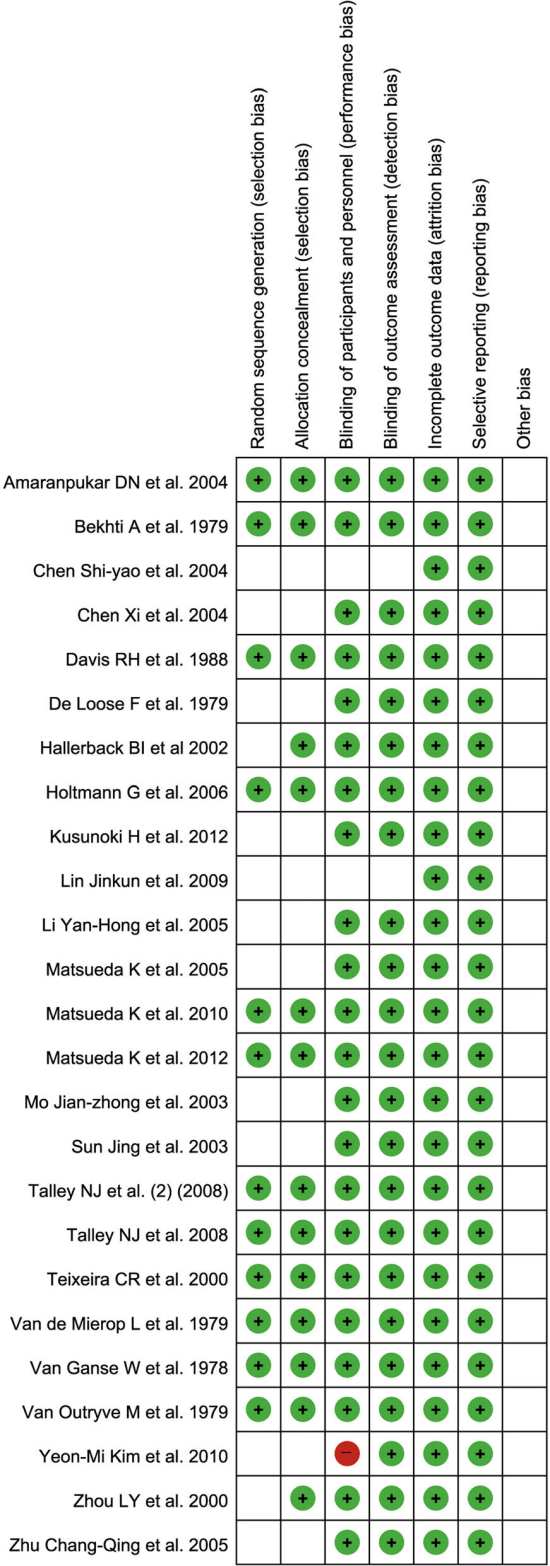
Risk of bias table of all enrolled studies. RoB, risk of bias. (+) denotes low risk of bias, blank denotes unclear risk of bias, (−) denotes high risk of bias

For the methodological quality of enrolled studies, the exact determination of random sequence generation and allocation concealment was not available, and double-blinding was not consistent in all of the enrolled studies. The summary of the risk of bias is demonstrated in Fig. 5, and the risk of bias table of all enrolled studies is shown in Fig. 6.

## Discussion

FD involves complex pathophysiologic mechanisms including visceral hypersensitivity, impaired gastric accommodation, delayed gastric emptying, *H. pylori* infection, psychosocial disorders, and even an unhealthy lifestyle [39–41]. The prevalence of gastric emptying delay was reported to be 37–39% in patients with FD. Consequently, prokinetics were developed based on the concept that promoting impaired gut motility could reduce the symptoms of FD [39, 40]. However, improved delayed gastric emptying was not associated with symptomatic relief in patients with gastroparesis [39, 40]. It is apparent that dysmotility cannot be the only target. Multiple other mechanisms should be considered in the treatment of FD.

Proton-pump inhibitors (PPI) and prokinetic agents are the mainstays of the treatment of FD [39], both of which have similar relative efficacy [42]. However, adverse events related to PPI have been reported, and the comparative efficacy of prokinetics has thus far not been evaluated [43, 44]. Because the adverse events associated with PPIs are linked to long-term use of this medication and the recurrence of FD is not infrequent [45], repeated prescriptions of PPI should be re-evaluated and additional focus should be directed toward the role of prokinetic agents in the treatment of FD.

In this analysis, the relative efficacy of prokinetics was based on the SUCRA value. However, there was no significant difference in efficacy between the prokinetics including metoclopramide, trimebutine, mosapride, and domperidone (Table 3). Though the mechanisms of action of these medications are slightly different, the precise reason for the superior or inferior comparative efficacy between these medications could not be evaluated in this analysis. For example, although mosapride, itopride, and acotiamide are all known to modulate gastric accommodation, the statistical effects of these drugs differed in this analysis [46–48].

The frequency of adverse event must also be considered in selecting prokinetics for the treatment of FD. In the case of metoclopramide, which is a central D_1_ and D_2_ receptor antagonist, extrapyramidal symptoms including dystonic movement or tardive dyskinesia, which are often irreversible, inhibit the administration of high doses or the long-term use of this medication [49].

In addition to the occurrence of adverse events, drug incompatibility due to drug interactions from a combination of prokinetics or other drugs should be considered in selecting prokinetics. Because domperidone, which is a peripheral D_2_ and D_3_ receptor antagonist is associated with ventricular arrhythmia, concomitant use of drugs that prolong the QTc interval, and potent CYP3A4 inhibitors should be avoided [50].

With respect to serotonergic agonists, targeting multiple receptors with non-selective inhibition can potentially lead to adverse events. Cardiac arrhythmia or QTc prolongation was most commonly associated with cisapride and tegaserod [51]. 5HT_1_ receptor subtypes have been suggested to account for adverse events due to interactions with HERG cardiac potassium channel and 5-hydroxytryptamine [52]. Mosapride, which is a nonselective 5-HT_4_ agonist with no HERG or 5HT_1_ affinity, could be substituted for metoclopramide or domperidone for the specific subset of patients who need long-term treatment.

Trimebutine, which is an enkephalin agonist, has a dual action on both of hyperkinetic and hypokinetic motility disorders [53]. This medication accelerates gastric emptying by inducing premature phase III activity of the migrating motor complex in gut [9]. Although most studies are focused on the treatment of irritable bowel syndrome, this medication could be substituted for other prokinetics that potentially have serious adverse effects.

In this study, we could not investigate the reason for the relatively lower efficacy of itopride, a mixed D_2_ receptor antagonist, and acetylcholinesterase inhibitor or acotiamide, an M_1_/M_2_ muscarinic receptor antagonist and acetylcholinesterase inhibitor. Given the probability of inconsistencies in the enrolled studies and the superior efficacy of prokinetics that have a relatively small number of enrolled population, there could be an overestimation of efficacy drawn from the pairwise indirect comparison.

This study is the first meta-analysis evaluating the comparative effectiveness of prokinetic agents. The strength of this study is the rigorous searching of the literature without language limitations and the use of an indirect comparative method to address the challenge of performing head-to-head analyses in clinical practice. Nonetheless, there are several limitations that impact the generalizability of the main results. All of the available prokinetic drugs could not be included in this study. These omitted agents include levosulpiride, a selective D_2_ receptor antagonist, erythromycin, a motilin receptor agonist, tandospirone, a 5HT_1A_ agonist, prucalopride, a selective 5HT_4_ agonist or DA-9701, a 5HT_4_ agonist, D_2_ receptor and 5HT_3_ antagonist. These medications are not available in some countries. Furthermore, levosulpiride is associated with drug-induced parkinsonism, inhibiting its wide application in clinical practice [54]. Prucalopride was developed and licensed for the treatment of constipation. Therefore, these drugs were excluded from the beginning of this study. Another limitation was the lack of data for some prokinetic agents in this analysis. The relatively small number of studies evaluating metoclopramide and trimebutine may in part explain the inconsistencies as well as the overestimation of the comparative effectiveness of some medications. In addition, most enrolled studies did not discriminate between epigastric pain and postprandial distress syndromes, which are subtypes of FD or post-infectious FD. Moreover, *H. pylori* infection, which is closely associated with the pathogenesis of FD, was not considered in most of the studies [39]. Overlap syndrome (FD + gastroesophageal reflux disease or FD + irritable bowel syndrome) which is frequently encountered in clinical practice, was also not considered [39]. A further limitation was the absence of a no common validated outcome measurement scale for all the enrolled studies. As we have noted in a previous study, there is no definite and unanimous way of defining symptomatic improvement in patients with FD [6]. Further studies that include newer prokinetics and control for the above-mentioned limitations are needed to confirm the results of this study.

## Conclusion

In conclusion, metoclopramide, trimebutine, mosapride, and domperidone showed better efficacy for the treatment of FD than that of itopride or acotiamide. Considering the adverse events related to metoclopramide or domperidone, the short-term use of these agents or the alternative use of trimebutine or mosapride could be recommended for the symptomatic relief of FD.

## Additional file

Additional file 1: Contains tables for data input form and initial data for the analysis of network meta-analysis. (XLSX 17.6 kb)

## Abbreviations

FD: Functional dyspepsia
OR: Odds ratio
PPI: Proton pump inhibitor
RCT: Randomized controlled trial
RoB: Risk of bias
SUCRA: Surface under the cumulative ranking curve
